# Greening and Land Surface Temperature in Newly Expanded and Old Built-Up Areas Across Urban Development Stages in the Yangtze River Delta

**DOI:** 10.3390/biology15181575

**Published:** 2026-09-08

**Authors:** Lina Zhang, Qingxian Wang, Yuanye Du, Zihan Liang, Fengqin Yan

**Affiliations:** 1 School of Public Health, Zhejiang Chinese Medical University, Hangzhou 310053, China; zhanglina@zcmu.edu.cn (L.Z.); 202311125811034@zcmu.edu.cn (Q.W.); 202212233605003@zcmu.edu.cn (Y.D.); 2 Zhejiang International Science and Technology Cooperation Base of Air Pollution and Health, Hangzhou 310053, China; 3 State Key Laboratory of Resources and Environmental Information System, Institute of Geographic Sciences and Natural Resources Research, Chinese Academy of Sciences, Beijing 100101, China; 4 The Second School of Clinical Medicine, Zhejiang Chinese Medical University, Hangzhou 310053, China; 202312214901007@zcmu.edu.cn

**Keywords:** vegetation, urban heat, built-up areas, urban development stage, Yangtze River Delta

## Abstract

Vegetation greenness helps cool cities, but how its cooling effect changes over time in newly expanded and existing old built-up areas (BUAs) across different urban development stages remains poorly understood. This study investigated the spatiotemporal trends of the Normalized Difference Vegetation Index (NDVI) and land surface temperature (LST) within newly expanded and old BUAs across cities of different development stages from 1992 to 2020. Utilizing the ESA CCI dataset for BUA delineation and Landsat-derived NDVI and LST, we applied the Mann–Kendall test and Sen’s slope estimator to analyze 26 core cities. City sizes were classified into five categories from small cities to super megacities based on their status, and then assigned to urban development stages ranging from Stage 4 (least developed) to Stage 1 (most developed). The results revealed a significant decrease in NDVI across all areas in both new BUA (average significant slope = −0.0009, *p* < 0.05) and old BUA (average significant slope = −0.0002, *p* < 0.05), and a significant increase in LST in both new BUA (average significant slope = 0.0818, *p* < 0.05) and old BUA (average significant slope = 0.0245, *p* < 0.05). In addition, NDVI trends in BUA expansion varied significantly across urban development stages (*p* for trend = 0.016). Excluding Stage 1 (Shanghai only), vegetation loss attenuated from the sharpest decline in Stage 4 (average significant slope = −0.0021, 36.60% significant, *p* < 0.05) to a moderate decline in Stage 3 (−0.0011, 26.03%), and reversed to a slight vegetation gain in Stage 2 (0.0031, 19.14%), whereas LST showed no significant stage-dependent pattern. These findings underscore the widespread decreasing NDVI and increasing LST in both newly expanded and old BUAs, with the stage-wise vegetation differences potentially reflecting varying policy and economic contexts.

## Simple Summary

Vegetation greenness mitigates urban heat; however, how its cooling effect evolves over time in newly expanded versus older built-up areas remains poorly understood. We quantified vegetation greenness indicated by the Normalized Difference Vegetation Index (NDVI) and land surface temperature (LST) in both newly expanded and older built-up areas across 26 cities in the Yangtze River Delta from 1992 to 2020. Across most cities, both newly expanded and older built-up areas exhibited declining vegetation greenness and rising temperatures. Meanwhile, new expansions warmed more intensely than older areas, as indicated by LST trends. Moreover, vegetation greenness trends varied markedly across urban development stages, with the most severe losses in the least developed stages (excluding Stage 1, Shanghai only) and relatively moderate declines or even slight improvements in the more advanced stages. These findings highlight a persistent trade-off between urban expansion and environmental quality, and suggest that protecting vegetation during critical growth phases may be a potential strategy for fostering healthier and more climate-resilient cities, pending validation through targeted intervention studies.

## 1. Introduction

Urban areas now accommodate over half of the global population, a proportion projected to reach 68% by 2050. China, as a rapidly urbanizing country in the 21st century, exemplifies this trend [1,2]. The Yangtze River Delta (YRD), in particular, has emerged as a quintessential region of intense development and a model for future urban planning [3,4]. However, this rapid urbanization has introduced significant challenges, including rising carbon emissions [5], more frequent extreme weather [6], the intensifying urban heat island effect [7], and increased heat-related mortality [8]. In this context, vegetation greenness serves as a critical indicator of a natural cooling solution, offering a multitude of benefits such as alleviation of urban heat islands [9,10] and heat-related disease burden [10]. Consequently, ensuring equitable access to urban green space (UGS) is a key priority of the 2030 Agenda for Sustainable Development. As one of China’s most significant contributors to national greening [11], the YRD presents a critical case for investigating how vegetation greenness and land surface temperature (LST) evolve amid urban expansion and within existing urban areas. However, with urban expansion slowing in many mature cities, the imperative has shifted from creating new green space to retrofitting existing built environments. To develop transferable models for such retrofitting, it is essential to understand the coupled dynamics of vegetation greenness and LST across new and old built-up areas (BUAs) at different developmental stages.

Previous research on vegetation greenness in BUAs has largely focused on the national or global scales post-2000, often centering on large or individual cities, with scant attention devoted to functional urban agglomerations. For instance, Moderate-resolution Imaging Spectroradiometer (MODIS) data (500 m resolution) were used to analyze 841 global cities from 2001 to 2018 [11], while the Global Human Settlement Layer (250 m) assessed the built-up growth in the European Union from 2000 to 2015 [12]. While some earlier work utilized high-resolution GF-1 imagery for Xiamen, China [13] or Landsat to examine Shenzhen’s urban–rural continuum [14], their single-city focus limits generalizability. Furthermore, while some studies have addressed BUA expansion [15], many have not concurrently analyzed the changes in vegetation greenness and LST within these areas, particularly the spatially uneven distribution of vegetation greenness amidst ongoing urbanization. Consequently, a significant gap remains in long-term (1990s to present), medium-to-high-resolution analyses that simultaneously track vegetation greenness and LST dynamics in both expanding and existing BUAs within the YRD, a region pivotal to China’s urbanization.

Moreover, several studies have linked vegetation changes to urbanization indicators (e.g., urban sprawl and impervious surfaces); however, the impact of different urban development stages remains insufficiently explored. For example, one study used real-time data to explore how urban sprawl affects green space accessibility [16]; this approach lacked long-term trend analysis. Another study of 318 Chinese cities (1990–2015) revealed uneven changes in the UGS/urban impervious surface (UIS) across city sizes but used decadal intervals and static city size classifications from 2015 [17], potentially overlooking dynamic, recent changes. An analysis of 320 Chinese cities identified the relationship between the urban population and UGS across five city sizes [18], yet included only a limited sample of YRD cities, failing to capture regional specificity. These national-scale studies are further complicated by inconsistent city size definitions and classification years, hindering the comparison between studies and a clear understanding of how vegetation and LST evolve at critical developmental stages. Therefore, there is a need for a dynamic framework that accounts for urban development stage transitions to better understand vegetation and LST changes during pivotal phases of urban growth.

Vegetation is widely recognized as a key strategy for mitigating urban heat. For instance, a study of 93 European cities suggested that increasing green infrastructure could prevent approximately 1.84% of all summer-related mortality [10]. In the YRD, a densely populated and rapidly developing economic hub, accelerated urbanization has not only reshaped the levels and distribution of vegetation greenness but has also intensified the heat stress. In 2023, the Chinese government issued “Notice on Deepening the Pilot Projects for Climate-Adaptive Urban Construction”, which sets the goal that cities at the prefecture level and above will have initiated climate-adaptive urban construction by 2035. However, it remains unclear how vegetation greenness influences LST within both newly expanded and old BUAs, which ignores the fundamental physical differences between newly constructed areas and aging urban cores. In addition, existing research on urban development stages primarily relies on fixed-time-point city size classification or classification by cross-sectional composite indices, which fails to capture the qualitative changes that occur when individual cities cross the State Council’s city-size classification thresholds. To bridge these gaps, this study investigates vegetation greenness and LST trends in both newly expanded and old BUAs across the YRD region from 1992 to 2020 using the Google Earth Engine (GEE) platform. Specifically, we introduced a dynamic stage-transition framework that tracked cities as they crossed official size thresholds, rather than relying on static classifications. We analyzed 26 core cities, utilizing the Mann–Kendall test and Sen’s slope estimator to analyze the annual BUA data (from the ESA CCI) and Landsat-derived Normalized Difference Vegetation Index (NDVI) and LST. City sizes are dynamically classified into five types (small cities to super megacities), with transitions categorized into four stages (Stage 4-1) between 1992 and 2020. This framework enables a detailed examination of NDVI and LST characteristics across different urban development stages. Our study addresses the following questions:

(1) Do NDVI and LST exhibit similar trends in the BUA expansion and the existing BUA of the 26 core YRD cities from 1992 to 2020?

(2) How do NDVI and LST trends in BUA expansion differ among cities undergoing different urban development stages?

The answers to these questions will provide urban policymakers with a deeper understanding of vegetation greenness and LST dynamics in both new and old urban areas, offering a scientific basis for shaping healthier and more sustainable urban environments.

## 2. Methods

### 2.1. Study Area

**Figure 1:**
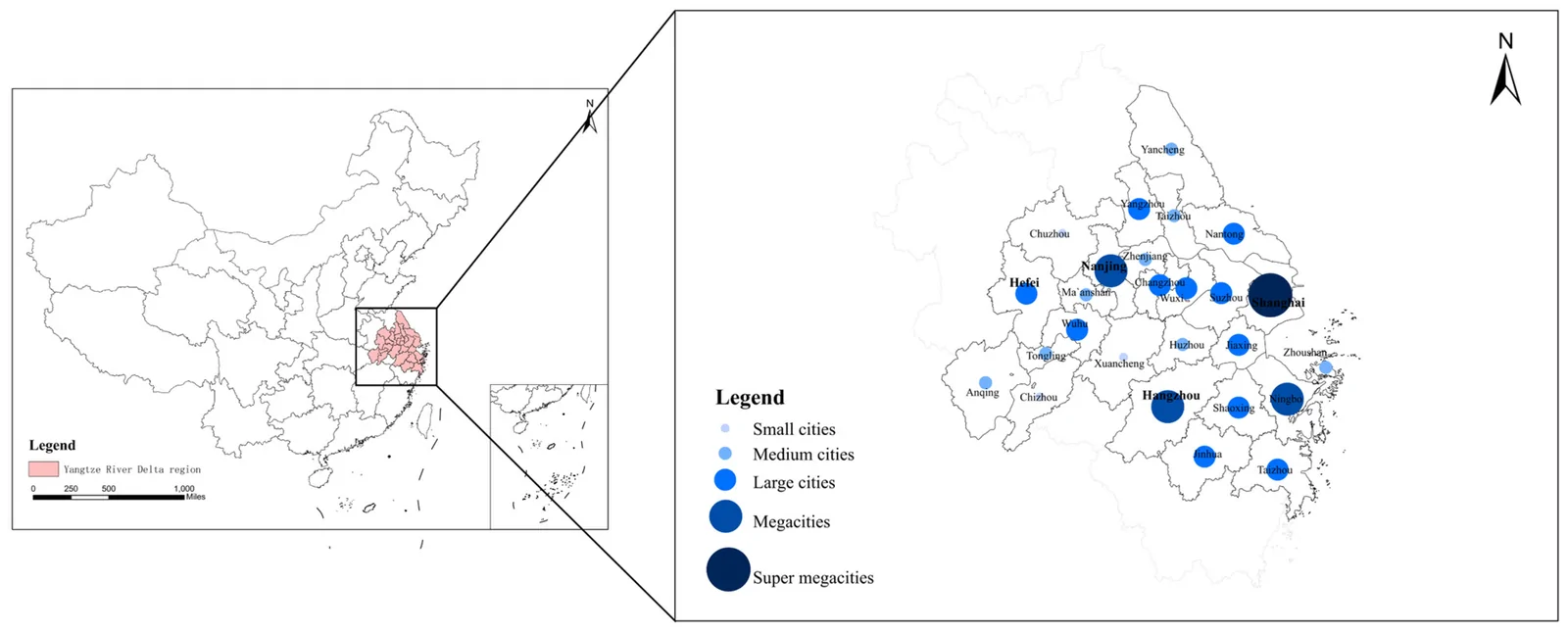
The locations and city sizes in 2020 of the 26 core cities in the Yangtze River Delta, China. Basemap source: Official 2019 China map (Survey and Review No. GS (2019) 1822), Ministry of Natural Resources of China.

The study focuses on the 26 core cities of the YRD region, a pivotal economic zone in China as delineated in the 2016 “Yangtze River Delta Urban Agglomeration Development” plan (Figure 1). Located in the lower Yangtze Plain, this area experiences a subtropical monsoon climate, which provides favorable conditions for urban development. The cities include Shanghai, eight from Zhejiang (Hangzhou, Ningbo, Shaoxing, Taizhou, Jiaxing, Jinhua, Huzhou, and Zhoushan), nine from Jiangsu (Suzhou, Nanjing, Wuxi, Nantong, Changzhou, Yancheng, Yangzhou, Zhenjiang, and Taizhou), and eight from Anhui (Hefei, Wuhu, Anqing, Ma’anshan, Chuzhou, Tongling, Chizhou, and Xuancheng). The study period of 1992–2020 was selected because it encompassed the most rapid and transformative phase of urbanization in the YRD, while ensuring continuous and consistent remote sensing data availability for robust trend analysis. Based on the State Council’s 2014 city size classification criteria and population data for 1992 and 2020, we categorized cities into five types: super megacities (>10 million), megacities (5–10 million), large cities (1–5 million), medium cities (0.5–1 million), and small cities (<0.5 million). The detailed classifications for 1992 and 2020 are provided in Table S1. Furthermore, based on theoretical foundations encompassing differential urbanization [19], migration-flow decomposition [20], and fractal hierarchy in rank-size distributions [21], we identified the years of development stage transitions from official government publications. These stage transitions capture qualitative shifts in growth trajectories when cities cross official thresholds, rather than merely describing their size at a fixed point in time. Specifically, seven cities transitioned from small to medium, twelve from medium to large, five from large to megacities, and one advanced from megacities to super megacities.

### 2.2. Data Sources and Processing

#### 2.2.1. Built-Up Area Data

The BUA distribution was primarily derived from the European Space Agency (ESA) Climate Change Initiative (CCI) Land Cover dataset. This dataset provides annual land cover classification at 300 m spatial resolution for the period of 1992–2020. BUA were identified using the classification code 190 within the ESA CCI legend. To maintain spatial consistency with other datasets used in this study, the original 300 m resolution data were resampled to 100 m resolution using a nearest-neighbor interpolation method. This dataset enabled the extraction of urban expansion patterns over the 1992–2020 timeframe, providing a long-term perspective on urbanization dynamics.

Urban expansion areas were identified by analyzing temporal changes in built-up classifications from this dataset. For each consecutive year pair, areas that transitioned from non-built-up to built-up categories were delineated as urban expansion. This resulted in the urban expansion chronosequences spanning 1992–2020 from ESA CCI data. The consistent 100 m spatial resolution enabled direct comparison and integration of expansion patterns.

#### 2.2.2. Vegetation Index

The NDVI data were derived from the Landsat series of satellites, including Landsat 5 Thematic Mapper (TM), Landsat 7 Enhanced Thematic Mapper Plus (ETM+), and Landsat 8 Operational Land Imager (OLI). These sensors provide consistent optical imagery at 30 m spatial resolution, suitable for vegetation monitoring.

Annual maximum NDVI composites were generated for each year from 1992 to 2020 to minimize atmospheric effects and cloud contamination. The maximum value composite approach selects the highest NDVI value for each pixel over a given year, representing the peak greenness during the growing season. To match the spatial resolution of the BUA dataset, the annual maximum NDVI composites were then resampled to 100 m resolution using a bilinear interpolation method. This consistent spatial framework enabled integrated analysis of urban expansion and vegetation dynamics.

#### 2.2.3. Land Surface Temperature

The long-term (1992–2020) LST dataset utilized in this study was derived from the Thermal Infrared Sensor (TIRS) and Thematic Mapper (TM/ETM+) instruments aboard the Landsat series (Landsat 5, 7, and 8), accessed via the GEE platform [22]. The Landsat Collection 2 Level-2 surface temperature products were utilized in this study, as atmospheric correction and LST retrieval have been completed by the USGS. Cloud, shadow, and snow masking were performed using the QA_PIXEL band derived from the CFMask algorithm [23], ensuring that only high-quality, clear-sky observations were retained for LST compositing.

Given that Landsat observations capture instantaneous LST at the time of spacecraft overpass (approximately 10:30 AM local solar time), temporal aggregation was required to match the annual granularity of the NDVI datasets. For each year, an annual median LST map was generated using a median value compositing technique during the peak growing season (June to September) [24,25].

### 2.3. Trend Analysis of Vegetation Changes and Urban Heat

The non-parametric Mann–Kendall (MK) trend test combined with Sen’s slope estimator was employed to analyze the spatial–temporal trends of NDVI and LST within urban areas. This approach is particularly suitable for environmental time series data as it does not require normally distributed data and is robust against outliers.

The Mann–Kendall test statistic S was calculated as follows:

(1) $$S={\Sigma_{i=1}}^{n-1}{\Sigma_{j=i+1}}^{n}sgn\left(x_{j}-x_{i}\right)$$

where n is the length of the time series, x_i_ and x_j_ are the sequential data values, and sgn() is the sign function.

The variance of S was computed as follows:

(2) $$\mathrm{Var}\left(S\right)=\frac{\left[n\left(n-1\right)\left(2n+5\right)-{\Sigma_{i=1}}^{m}t_{i}\left(t_{i}-1\right)\left(2t_{i}+5\right)\right]}{18}$$

where m is the number of tied groups and t_i_ is the number of data points in the i-th tied group.

The magnitude of trends was quantified using Sen’s slope estimator, which calculates the median of all possible pairwise slopes in the time series:

(3) $$\beta =\mathrm{median}\left[\frac{x_{j}-x_{i}}{j-i}\right]\mathrm{for}\;\mathrm{all}\;i<j$$

where β represents the annual rate of change in NDVI or LST, and x_i_ and x_j_ are values at times i and j, respectively.

The significance of trends was assessed using a two-tailed test based on the Z statistic. A significance level of *p* < 0.05 was applied to identify statistically significant trends. *p*-values were calculated as follows:

(4) $$p=2\times \left[1-\Phi \left(\left|Z\right|\right)\right]$$

where Φ is the standard normal cumulative distribution function.

This trend analysis was implemented on a pixel-by-pixel basis across all urban areas. For each pixel, the complete NDVI time series (1992–2020) underwent the MK test and Sen’s slope estimation. Moreover, we evaluated temporal autocorrelation using the lag-1 autocorrelation function (ACF) and Durbin–Watson statistics for both NDVI and LST time series (1992–2020). This approach generated two primary output layers: (1) a slope map representing the magnitude and direction of NDVI and LST changes; (2) a significance map indicating the statistical reliability of detected trends. The pixel-based methodology enabled detailed spatial analysis of vegetation dynamics within different urban contexts and expansion chronologies. Given the subtropical monsoon climate of the YRD, water availability is generally not the primary limiting factor for vegetation growth. Consequently, the long-term trends derived from annual maximum NDVI and growing-season median LST composites primarily reflect urbanization-driven land-use changes rather than meteorological drought or phenological modulation. Furthermore, to examine the differences in NDVI and LST trends across provinces, the Kruskal–Wallis H-test was employed at city level values. Due to limited sample availability (n = 1), Shanghai was excluded from the provincial-stratified comparison. Moreover, although some cities experienced multiple consecutive stage transitions during the study period (1992–2020), the NDVI and LST trend analyses for each stage were based on strictly non-overlapping time periods. Given the ordinal nature of urban development stages, the Jonckheere–Terpstra test was employed to detect trends [26]. Additionally, to address potential non-independence arising from repeated cities with multiple stage transitions, a sensitivity analysis restricted to non-repeated city-stage events was performed. Because some cities with multiple stage transitions were involved in Stage 3, their Stage 3 records were removed to balance sample sizes across stages.

### 2.4. Quantitative Relationship Between NDVI and LST

To quantitatively evaluate the coupled dynamics of NDVI and LST, a pixel-wise Spearman correlation analysis was performed between the annual NDVI and LST time series over the 1992–2020 period. For each built-up pixel, the correlation coefficient and its statistical significance (two-tailed test, α = 0.05) were calculated. This analysis was restricted to the built-up areas as of 2020.

## 3. Results

### 3.1. Trends of BUA Expansion and NDVI and LST in Newly Expanded BUAs over the Past Three Decades

**Figure 2:**
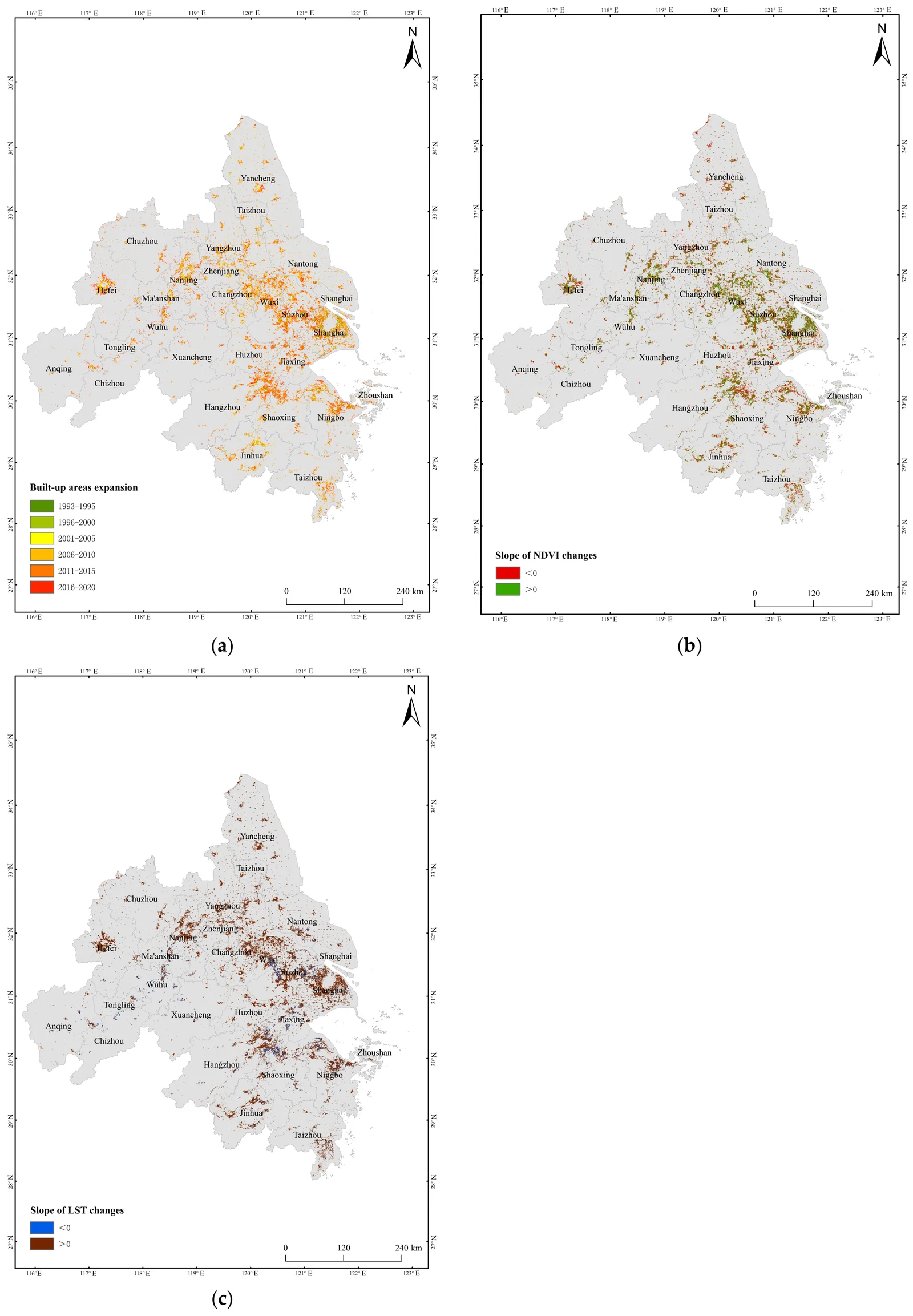
(**a**) Spatial distribution of BUA expansion across the 26 core cities in the YRD from 1992 to 2020; (**b**) the trends of NDVI in BUA expansion from 1992 to 2020; (**c**) and the trends of LST in BUA expansion from 1992 to 2020.

Figure 2 illustrates the spatial distribution of BUA expansion across the 26 core cities in the YRD from 1992 to 2020, along with the spatial patterns of NDVI and LST trends within the expanded BUA over the same period. A consistent increase in LST was observed within urban expansion areas across these cities. As summarized in Table 1, the average NDVI and LST slopes across all cities were −0.0010 and 0.0494, respectively. Regionally, the significant average slopes of NDVI and LST were 0.0023 and 0.0631 for Shanghai, −0.0007 and 0.0911 for Zhejiang, −0.0009 and 0.0765 for Jiangsu, and −0.0014 and 0.0806 for Anhui, with all provinces exhibiting statistically significant LST increases indicative of urban warming. Notably, while LST exhibited significant inter-provincial differences in warming intensity (*p* = 0.018), the proportion of significantly warming pixels was not statistically significant across provinces (*p* = 0.234). Conversely, excluding Shanghai, all provinces showed declining NDVI trends, though the inter-provincial difference in the significant average slope did not reach statistical significance; the proportion of pixels with a significant NDVI change differed markedly among provinces (*p* = 0.019). Specifically, the average proportions of significant changes in NDVI and LST across the 26 core cities were 21.40% and 20.66%, respectively. At the city level, 10 of the 26 cities exhibited significantly increasing NDVI trends, such as Shanghai, Hangzhou, and Nanjing (Table S2). In addition, temporal autocorrelation analysis showed negligible autocorrelation for LST (median ACF = 0.064), satisfying the MK independence assumption. For NDVI, the moderate autocorrelation (median ACF = 0.328) was reduced to 0.251 after Sen’s slope detrending; this weak residual autocorrelation supported the validity of the standard MK test.

### 3.2. Changes in NDVI and LST in Older BUAs over the Past Three Decades

**Figure 3:**
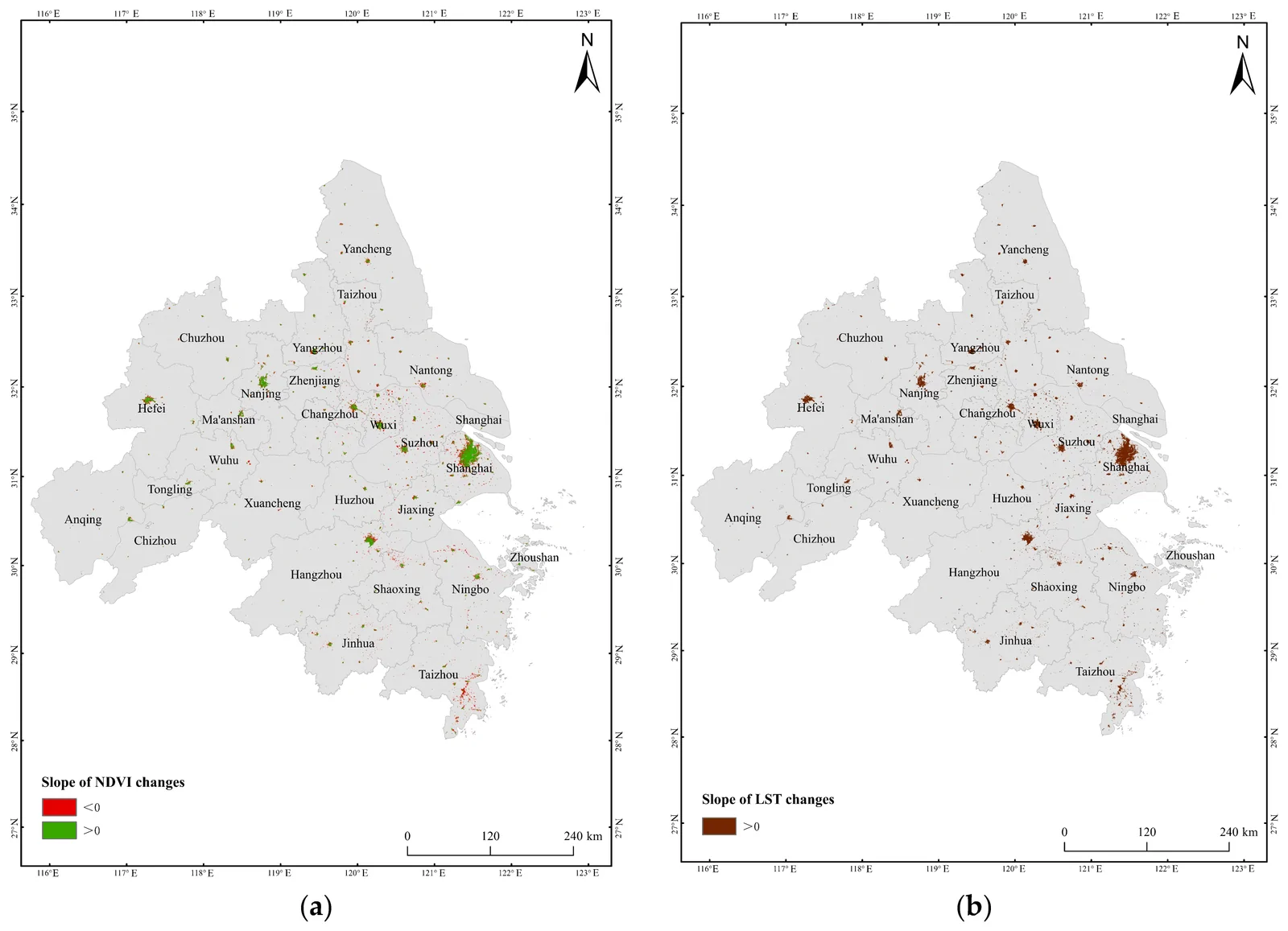
Spatial distribution of the trends of (**a**) NDVI and (**b**) LST in old BUAs in the YRD region from 1992 to 2020.

Between 1992 and 2020, LST within older BUAs also showed an overall increase, with a significant average slope of 0.0245 across the 26 cities (Table 2). Regionally, the significant average slopes of LST were 0.0261 for Shanghai, 0.0264 for Zhejiang, 0.0235 for Jiangsu, and 0.0216 for Anhui. Both the average slope and the significant average slope of NDVI and LST exhibited significant inter-provincial differences in older BUAs; however, the proportion of significantly warming pixels did not differ significantly across provinces (*p* = 0.063), despite Anhui showing a markedly lower percentage. A city-level analysis further indicated that all cities experienced significant LST increases in older BUAs (Table S3). The average proportions of significant changes in NDVI and LST across the 26 core cities were 58.29% and 46.24%, respectively. Spatially, NDVI in older BUAs showed divergent trends among provinces (Figure 3). Notably, despite the significant inter-provincial difference in the average NDVI slope, the proportion of pixels with a significant NDVI change was not statistically significant across provinces (*p* = 0.389). Furthermore, to further examine the quantitative relationship between vegetation and surface temperature within the BUA, a pixel-wise Spearman correlation analysis was conducted. The mean Spearman correlation coefficient between annual NDVI and LST from 1992 to 2020 within the built-up areas of 2020 was −0.13. The majority of pixels (87.27%) showed statistically insignificant correlations (Figure S1). Among the 12.73% of pixels with significant correlations, 95.1% exhibited negative coefficients.

### 3.3. Disparities in NDVI and LST Trends Across Urban Development Stages in BUA Expansion

The NDVI trends in BUA expansion varied markedly across different urban development stages (*p* for trend = 0.016, 0.016, and 0.035 for the NDVI average slope, significant slope, and proportion of significant change, respectively; Table 3). Specifically, NDVI losses attenuated progressively from Stage 4 to Stage 2: the average slopes increased from −0.0014 (Stage 4) to −0.0006 (Stage 3) and turned slightly positive in Stage 2 (0.0004), while the significant slopes followed a comparable trajectory from −0.0021 to −0.0011 to 0.0031, excluding Stage 1 (n = 1). However, LST trends during BUA expansion were less consistent across urban development stages, with no significant trends observed across stages (Table 3). The sensitivity analysis, which excluded repeated cities across different stage transitions, was robust and confirmed that the trends remained consistent (Table S4).

## 4. Discussion

### 4.1. Developments in NDVI and LST in BUA Expansion and Older BUAs over the Past Three Decades

Our analysis over the past three decades showed that, within the 26 core cities of the YRD, both newly expanded and older BUAs mainly experienced reductions in vegetation greenness alongside an urban warming trend. This pattern was consistent with global urbanization trends. A national study of 328 cities (1990–2022) identified a pivotal shift around 2005, transitioning from a slight decline in fractional vegetation cover (FVC) before 2005 to a widespread increase thereafter, with notably higher FVC levels in new urban areas compared to older ones [27]. Similarly, another national study (2000–2020) highlighted a significant overall UGS loss in China, more acute in eastern cities [18]. Another study of 318 Chinese cities (1990–2015), including key YRD cities like Shanghai, Nanjing, and Hangzhou, documented concurrent rises in both UIS and UGS, especially during 2000–2010 [17]. However, many previous studies relying on 5- or 10-year intervals lack the temporal resolution to capture annual vegetation dynamics, and those initiated post-2000 may miss longer-term historical trends. A global analysis (2001–2018) of 841 cities reported sustainable greening alongside BUA expansion [11]. These apparent differences underscore the necessity of long-term, region-specific analyses. Major urban construction in many areas predates 2001, and broad-scale studies can mask regional patterns. Therefore, our long-term study of the YRD, a prime example of rapid economic growth, provided crucial insights into vegetation greenness changes.

However, several cities exhibited patterns that diverged from the regional trend. In newly expanded BUAs, although the overall NDVI trend was negative across the YRD, some cities (e.g., Shanghai, Hangzhou, and Nanjing) showed increasing vegetation greenness. This difference may reflect differentiated urbanization pathways. Economically advanced coastal cities have generally reached later stages of urbanization, where stronger fiscal capacities could support greater investment in urban greening during new development. For instance, Shanghai enacted the Greening Regulations of Shanghai Municipality in 2007, which mandated minimum green space ratios in construction projects and coincided with the vegetation gains in urban areas [28]. Hangzhou’s spatial planning frameworks (e.g., the “Nuclear, Belts, or Banks” strategy) have reportedly prioritized green infrastructure in expansion zones. By contrast, cities in central and northern Anhui (e.g., Xuancheng, Chuzhou, and Anqing) recorded the steepest NDVI declines in expansion areas, consistent with their status as industrialization-driven cities where rapid land conversion for manufacturing occurred [17]. In older BUAs, the difference was equally pronounced. While the total regional average NDVI slope was slightly negative, multiple cities in Anhui (e.g., Hefei, Wuhu, Ma’anshan, Anqing, Chuzhou, Tongling, and Chizhou) exhibited positive greenness trends. Concurrently, LST in older BUAs rose most modestly in Anhui compared with Shanghai, Zhejiang, and Jiangsu. Overall, the NDVI and LST patterns in newly expanded and old BUAs varied markedly across cities. Moreover, urban heat was influenced by multiple factors, including land use (e.g., farmland and wetland), meteorological conditions (e.g., precipitation and air temperature), and anthropogenic activities (e.g., transport and industry) [29]. Thus, clarifying the relationship between vegetation greenness and LST in both newly expanded and older BUAs should be an urgent priority for future research to guide the effective development of climate-adaptive cities.

### 4.2. NDVI and LST Trends Across Different Urban Development Stages

Our study demonstrated that, excluding Stage 1 (Shanghai only; n = 1), NDVI trends during BUA expansion varied significantly across urban development stages, with the sharpest decline recorded in Stage 4, a moderate loss in Stage 3, and a slight increase in Stage 2. By contrast, LST trends showed no significant stage-dependent patterns. Notably, given that a subset of cities was represented across multiple stages, stage-wise comparisons should be interpreted cautiously; nevertheless, the sensitivity analysis supports the robustness of these patterns under conditions of reduced city-level dependence. Direct evidence linking urban development transitions to NDVI and LST trends remains scarce, as prior research often uses static size classifications from disparate years, inconsistent population definitions, or composite indicators to define development stages. Nevertheless, some consistent patterns emerged. For example, the influence of vegetation greenness on the UHI intensified with the urban development stage [30]. Meanwhile, cities at the urban maturity stage demonstrated an enhanced capacity to regulate their local climate [31]. In addition, research based on 2015 city sizes found that megacities (≥10 million) experienced the most rapid BUA expansion from 1990 to 2000, while eastern large (5–10 million) and medium-sized (1–5 million) cities led expansion from 2000 to 2010; concurrently, megacities consistently maintained the highest UGS proportion [17]. Similarly, an analysis of 328 cities (1990–2022) showed megacities achieved the most rapid FVC increase after 2005 [27]. Conversely, another study reported that medium-sized cities (500,000–1 million) experienced the most rapid loss of UGS, though megacities (>5 million) led in UGS coverage after 2015 [18]. These inconsistent findings primarily result from differences in study periods, city size definitions, and baseline years, highlighting the critical need for a standardized transition-based analysis, such as that employed here, to understand vegetation greenness and LST dynamics at critical urban development stages.

The tendency toward reduced vegetation losses or even slight greening in more developed cities is consistent with the hypothesis that a stronger policy and economic capacity are associated with urban greening in the 21st century. Stage 1 was an exception, as it comprised only Shanghai. Having completed its transition to a super megacity by 2001, Shanghai’s early transformation predated the intensive rollout of national ecological policies after 2000, including the 2015 “Accelerating the Promotion of the Construction of an Ecological Civilization” and local greening strategies such as the “Nuclear, Belts, or Banks” planning in Hangzhou and Nanjing. Its rapid urbanization during 1992–2001 might have proceeded independently of the policy-driven vegetation protection measures. Previous studies suggested that economic strength has been associated with megacities’ investment in ecological infrastructure to mitigate urban heat [17,27]. Specifically, robust economic development, typically indicated by urban GDP, has been reported to be strongly associated with greening efforts [27]. Meanwhile, it has been argued that cities increasingly recognize the multifaceted benefits of vegetation greenness in enhancing public health, bolstering city branding and economies, and supporting ecological construction [17]. Accordingly, urban development stage transitions could represent windows for integrating advanced vegetation greenness planning, particularly in less developed urban areas.

### 4.3. Mechanisms Underlying Greenness and LST Trends in Newly Expanded and Older BUAs

Both newly expanded and older BUAs in most cities exhibited a concurrent NDVI decline and LST rise, rooted in altered surface energy partitioning as impervious surfaces replace vegetation, diverting more absorbed radiation into sensible heat and subsurface storage at the expense of latent cooling [32,33]. Notably, new BUAs warmed markedly faster than older BUAs, suggesting an abrupt biophysical shift that accompanies the first phase of urban land conversion. Conversely, cities at advanced development stages (e.g., Shanghai) observed an NDVI rebound and relatively moderated LST growth during expansion, suggesting that strategic vegetation layout and patch connectivity could revive evaporative cooling, enhance latent heat loss, and curb thermal buildup [32]. Collectively, declining green cover likely promoted surface warming by constraining evapotranspiration and the associated latent heat flux, whereas mature cities might theoretically re-establish biophysical cooling through development-stage-sensitive planning that refines vegetation greenness placement.

### 4.4. Implications for Urban Planning and Policy

Understanding the divergent vegetation greenness and LST trends in new versus older BUAs and their relationship to stage transitions is vital for fostering healthy urban environments and guiding sustainable development. Vegetation greenness is well-documented for providing critical ecosystem services [34] and enhancing public health and well-being [35,36]. Our findings, suggesting more pronounced vegetation loss and intensified warming in newly expanded BUAs, may help identify precise spatial targets for urban warming mitigation. The World Health Organization (WHO) recommends universal access to at least 0.5 hectares of green space within a 300 m linear distance of residences [37]. However, significant gaps persist; over 60% of the urban population in numerous European and Chinese cities still lived in areas with insufficient green space coverage [35,38]. This deficit suggests a need for urban policymakers to consider prioritizing vegetation greenness integration in the redevelopment of older BUAs. Given that smaller green space, such as street trees, can constitute over one-third of the total UGS and are crucial for connectivity [39], planners could consider prioritizing the integration of fragmented green space into older urban fabrics [40]. Furthermore, as cities incorporate more vegetation greenness during transitions to larger sizes, development stages could inform strategies to strategically enhance green infrastructure, making vegetation greenness enhancement an important component of healthy city strategies, especially for smaller or growing cities.

### 4.5. Limitations and Future Research

This study employed the 30 m resolution Landsat archive to derive NDVI for vegetation greenness over a 30-year period, enabling a comprehensive long-term trend analysis. However, this spatial resolution may fail to capture very small greening areas. Additionally, NDVI serves as a proxy for greenness rather than specific vegetation types. It cannot distinguish between vegetation structures (e.g., trees vs. grass) or functional roles (e.g., park vs. agricultural land). Future research could employ higher-resolution imagery to enhance the accuracy of vegetation greenness and land-use type extraction and classification, though this must be balanced against temporal depth and coverage. In addition, all datasets were resampled to a uniform 100 m grid. This process may introduce scale-induced uncertainties: the 300 m CCI data imposed its coarser spatial structure onto the 100 m grid, while the 100 m resampling of 30 m Landsat data inevitably smoothed the fine-scale spatial heterogeneity. Moreover, city size classification was primarily based on official government publications, which occasionally presented inconsistencies with statistical yearbooks; in such cases, official releases were considered authoritative. Although strictly non-overlapping periods may not guarantee full statistical independence because some cities appear in multiple stages, our sensitivity analysis, which removed the Stage 3 records of repeated cities, supported the robustness of the findings. Finally, while our sample included only one super megacity transition (Shanghai), future studies should incorporate more transitions to super megacity status to enhance the statistical robustness and generalizability of findings related to the highest level of urban transition.

## 5. Conclusions

This study examined the spatiotemporal dynamics of NDVI and LST in 26 core cities of the YRD region over the past three decades. The findings indicated a decreasing NDVI and increasing LST in most newly expanded and older BUAs, with more pronounced warming in new expansions. NDVI trends in BUA expansion varied significantly across urban development stages. Excluding Stage 1 (Shanghai only; n = 1), vegetation loss attenuated progressively from Stage 4 (sharpest) to Stage 3 (moderate) to Stage 2 (slight increase), whereas LST showed no significant stage-dependent pattern. These stage-wise differences may be associated with varying policy and economic contexts, though such links need to be directly quantified in future research. Limitations included the 30 m resolution of Landsat data, the use of NDVI as a general greenness proxy without distinguishing between vegetation types, and the small sample size for Stage 1. Future work should incorporate higher-resolution imagery, object-based vegetation classification, and additional super megacities to strengthen our conclusions. Based on the observed patterns, prioritizing vegetation greenness protection during early urban expansion phases, particularly in smaller cities, could serve as a potential strategy for sustainable urban planning, pending validation by future studies that directly assess policy effectiveness.

## Figures and Tables

**Table 1:** Changes in NDVI and LST in BUA expansion in the YRD region from 1992 to 2020.

City	Average Slope		Significant Average Slope		Proportion of Significant Change (%)	
	NDVI	LST	NDVI	LST	NDVI	LST
All areas	−0.0010	0.0494	−0.0009	0.0818	21.40	20.66
Shanghai	0.0004	0.0400	0.0023	0.0631	30.11	16.65
Zhejiang	−0.0009	0.0600	−0.0007	0.0911	24.00	16.77
Jiangsu	−0.0005	0.0539	−0.0009	0.0765	20.64	27.61
Anhui	−0.0017	0.0349	−0.0014	0.0806	18.57	17.23
*p* *	0.209	0.011	0.611	0.018	0.019	0.234

Notes: Average slope: the mean value of Sen’s slope estimates for all pixels in the analysis region, representing the overall rate of change in NDVI and LST over the study period. Significant average slope: the mean value of Sen’s slope estimates only for pixels that passed the significance test based on the Mann–Kendall test (*p* < 0.05). *p* * presents the comparison among Zhejiang, Jiangsu, and Anhui provinces (Shanghai excluded).

**Table 2:** Changes in NDVI in old BUA in the YRD region from 1992 to 2020.

City	Average Slope		Significant Average Slope		Proportion of Significant Change (%)	
	NDVI	LST	NDVI	LST	NDVI	LST
All areas	−0.0002	0.0224	−0.0002	0.0245	58.29	46.24
Shanghai	0.0009	0.0258	0.0014	0.0261	64.94	83.08
Zhejiang	−0.0008	0.0256	−0.0011	0.0264	60.72	73.17
Jiangsu	−0.0003	0.0226	−0.0005	0.0235	56.76	58.06
Anhui	0.0004	0.0187	0.0007	0.0216	56.73	23.08
*p* *	0.019	<0.001	0.011	0.027	0.389	0.063

Notes: Average slope: the mean value of Sen’s slope estimates for all pixels in the analysis region, representing the overall rate of change in NDVI and LST over the study period. Significant average slope: the mean value of Sen’s slope estimates only for pixels that passed the significance test based on the Mann–Kendall test (*p* < 0.05). *p* * presents the comparison among Zhejiang, Jiangsu, and Anhui provinces (Shanghai excluded).

**Table 3:** The trend of changes in NDVI and LST during BUA expansion across different urban development stages.

Stage	Cities	Average Slope		Average Significant Slope		Proportion of Significant Change (%)	
		NDVI	LST	NDVI	LST	NDVI	LST
Stage 1 *	1	−0.0017	0.0199	−0.0028	0.0252	46.53	1.44
Stage 2 *	5	0.0004	0.0473	0.0031	0.0857	19.14	13.75
Stage 3 *	12	−0.0006	0.0573	−0.0011	0.0810	26.03	24.73
Stage 4 *	7	−0.0014	0.0370	−0.0021	0.0581	36.60	26.58
*p* for trend		0.016	0.266	0.016	0.101	0.035	0.726

* Urban development stages are defined as follows: Stage 1 * represents the transition from megacities to super megacities, comprising Shanghai. Stage 2 * denotes the transition from large cities to megacities, including Hangzhou, Ningbo, Nanjing, Nantong, and Wuxi. Stage 3 * signifies the transition from medium to large cities, encompassing Ningbo, Nantong, Wuxi, Jinhua, Taizhou (Zhejiang), Yancheng, Jiaxing, Shaoxing, Changzhou, Yangzhou, Taizhou (Jiangsu), and Wuhu. Stage 4 * captures the transition from small to medium cities, involving Jinhua, Yancheng, Jiaxing, Taizhou (Jiangsu), Huzhou, Ma’anshan, and Tongling. Note that some cities appear in multiple stages as they underwent consecutive transitions. Average slope: the mean value of Sen’s slope estimates for all pixels in the analysis region. It represents the overall average rate of change in NDVI and LST over the study period; significant average slope: the mean value of Sen’s slope estimates only for pixels that passed the significance test (*p* < 0.05 in the Mann–Kendall test).

## Data Availability

The raw data supporting the conclusions of this article will be made available by the authors upon request.

## Supplementary Materials

The following supporting information can be downloaded at: https://www.mdpi.com/article/10.3390/biology15181575/s1, Table S1: Classification of the sizes for the 26 core cities in the YRD region in 1992 and 2020; Table S2: Changes in NDVI and LST in BUA expansion in the YRD region from 1992 to 2020 for each city; Table S3: Changes in NDVI and LST in old BUA in the YRD region from 1992 to 2020 for each city; Table S4: Sensitivity analysis of NDVI and LST trends during BUA expansion across different urban development stages, excluding repeated city-stage transitions; Figure S1: Spatial distribution of Spearman correlation coefficients between annual NDVI and LST in the Yangtze River Delta: (a) correlation coefficient; and (b) statistical significance (two-tailed test, α = 0.05).

## Abbreviations

The following abbreviations are used in this manuscript:

NDVI	Normalized Difference Vegetation Index
LST	land surface temperature
BUAs	built-up areas
ESA	European Space Agency
CCI	Climate Change Initiative
