# Supplementary material for: Greening and Land Surface Temperature in Newly Expanded and Old Built-Up Areas Across Urban Development Stages in the Yangtze River Delta
Source: Biology (Basel). 2026 Sep 8;15(18):1575. doi: 10.3390/biology15181575 (PMC13603347; doi:10.3390/biology15181575)
Supplement: Supplementary file 1 [file biology-15-01575-s001.zip › biology-4541577-supplementary.pdf]

biology

MDPI

Supplementary Materials

Greening and land surface temperature in newly expanded and old built-up areas across urban development stages in the Yangtze River Delta

Lina Zhang [1,2,3,†], Qingxian Wang [1,†], Yuanye Du [1], Zihan Liang [4] and Fengqin Yan [3,*]

[1] School of Public Health, Zhejiang Chinese Medical University, Hangzhou 310053, China; zhanglina@zcmu.edu.cn (L.Z.); 202311125811034@zcmu.edu.cn (Q.W.); 202212233605003@zcmu.edu.cn (Y.D.)

[2] Zhejiang International Science and Technology Cooperation Base of Air Pollution and Health, Hangzhou 310053, China

[3] State Key Laboratory of Resources and Environmental Information System, Institute of Geographic Sciences and Natural Resources Research, Chinese Academy of Sciences, Beijing, 100101, China

[4] The Second School of Clinical Medicine, Zhejiang Chinese Medical University, Hangzhou 310053, China; 202312214901007@zcmu.edu.cn

* Correspondence: yanfq@lreis.ac.cn

† These authors contributed equally to this work.

Table of Contents

Tabel S1: Classification of the sizes for the 26 core cities in the YRD region in 1992 and 2020;
Table S2: Changes of NDVI an LST in BUA expansion in the YRD region from 1992 to 2020 for each city;
Table S3: Changes of NDVI and LST in old BUA in the YRD region from 1992 to 2020 for each city;
Table S4: Sensitivity analysis of NDVI and LST trends during BUA expansion across different urban development stages, excluding repeated city-stage transitions;
Figure S1: Spatial distribution of Spearman correlation coefficients between annual NDVI and LST in the Yangtze River Delta. (a) Correlation coefficient; (b) statistical significance (two-tailed test, α = 0.05).

Tabel S1. Classification of the sizes for the 26 core cities in the YRD region in 1992 and 2020.

Province	City	Classification in 1992	Classification in 2020
Shanghai	Shanghai	Megacities	Super megacities*
Zhejiang	Hangzhou	Large cities	Megacities
	Ningbo	Medium cities	Megacities*
	Shaoxing	Medium cities	Large cities*
	Taizhou	Medium cities	Large cities
	Jinhua	Small cities	Large cities
	Jiaxing	Small cities	Large cities
	Huzhou	Small cities	Medium cities*
	Zhoushan	Medium cities	Medium cities
Jiangsu	Nanjing	Large cities	Megacities
	Suzhou	Large cities	Large cities
	Wuxi	Medium cities	Large cities
	Nantong	Medium cities	Large cities
	Changzhou	Medium cities	Large cities
	Yangzhou	Medium cities	Large cities
	Yancheng	Small cities	Medium cities
	Zhenjiang	Medium cities	Medium cities
	Taizhou	Small cities	Medium cities
Anhui	Hefei	Large cities	Large cities
	Wuhu	Medium cities	Large cities
	Ma'anshan	Small cities	Medium cities
	Anqing	Medium cities	Medium cities
	Chuzhou	Small cities	Small cities *
	Xuancheng	Small cities	Small cities
	Tongling	Small cities	Medium cities
	Chizhou	Small cities	Small cities

***Super megacities** (＞10 million); **megacities** (5-10 million); **large cities** (1-5 million); **medium cities** (0.5-1 million); **small cities** (＜0.5 million).

Tabel S2. Changes of NDVI an LST in BUA expansion in the YRD region from 1992 to 2020 for each city.

City	Average slope		Significant average slope		Proportion of significant change (%)	
	NDVI	LST	NDVI	LST	NDVI	LST
Shanghai	0.0004	0.0400	0.0023	0.0631	30.11	16.65
Hangzhou	0.00001	0.0508	0.0003	0.0806	23.49	1.32
Ningbo	-0.0012	0.0522	-0.0008	0.0941	22.83	13.79
Shaoxing	-0.0010	0.0466	-0.0014	0.0892	22.20	3.22
Taizhou	-0.0015	0.0811	-0.0026	0.1121	22.53	37.52
Jinhua	-0.0011	0.0536	-0.0010	0.0941	29.58	23.46
Jiaxing	-0.0023	0.0420	-0.0032	0.0703	20.17	2.14
Huzhou	-0.0026	0.0624	-0.0026	0.0881	21.29	11.50
Zhoushan	0.0025	0.0915	0.0055	0.1003	29.87	41.24
Nanjing	-0.0005	0.0431	0.0017	0.0695	23.33	20.24
Suzhou	0.0003	0.0398	0.0010	0.0749	20.19	16.16
Wuxi	0.0012	0.0411	0.0019	0.0657	21.96	25.87
Nantong	-0.0002	0.0506	0.00002	0.0732	18.49	32.18
Changzhou	0.0003	0.0443	0.0010	0.0653	22.37	14.61
Yangzhou	-0.0023	0.0727	-0.0049	0.0867	20.87	34.88
Yancheng	-0.0019	0.0679	-0.0046	0.0851	18.63	34.68
Zhenjiang	-0.0003	0.0551	-0.0001	0.0819	22.58	32.69
Taizhou	-0.0008	0.0703	-0.0047	0.0865	17.34	37.21
Hefei	-0.0021	0.0456	-0.0021	0.0693	19.05	19.19
Wuhu	-0.0002	0.0288	0.0031	0.0781	19.98	20.14
Ma'anshan	0.0007	0.0350	-0.0002	0.0730	21.75	14.80
Anqing	-0.0027	0.0277	-0.0053	0.0902	20.70	17.03
Chuzhou	-0.0032	0.0767	-0.0033	0.0796	15.29	21.92
Xuancheng	-0.0042	0.0279	-0.0035	0.0820	16.46	15.15
Tongling	-0.0010	0.0203	0.0025	0.0839	20.23	15.84
Chizhou	-0.0010	0.0171	-0.0023	0.0886	15.13	13.73

Tabel S3. Changes of NDVI and LST in old BUA in the YRD region from 1992 to 2020 for each city.

City	Average slope		Significant average slope		Proportion of significant change (%)	
	NDVI	LST	NDVI	LST	NDVI	LST
Shanghai	0.0009	0.0258	0.0014	0.0261	64.94	83.08
Hangzhou	-0.00003	0.0213	0.00001	0.0283	65.93	5.94
Ningbo	-0.0009	0.0252	-0.0013	0.0252	59.10	99.35
Shaoxing	-0.0015	0.0242	-0.0021	0.0246	64.95	75.26
Taizhou	-0.0023	0.0290	-0.0034	0.0290	64.42	100
Jinhua	-0.0009	0.0315	-0.0014	0.0315	56.53	100
Jiaxing	-0.0007	0.0254	-0.0009	0.0255	58.51	93.10
Huzhou	-0.0004	0.0223	-0.0006	0.0215	51.43	13.91
Zhoushan	0.0007	0.0256	0.0010	0.0257	64.86	97.83
Nanjing	0.0009	0.0194	0.0014	NA	61.33	0
Suzhou	-0.0013	0.0235	-0.0020	0.0243	60.09	51.75
Wuxi	-0.0001	0.0223	-0.0001	0.0228	63.42	14.26
Nantong	-0.0007	0.0245	-0.0011	0.0249	53.41	79.84
Changzhou	-0.0003	0.0222	-0.0004	0.0223	58.04	67.20
Yangzhou	0.0003	0.0220	0.0006	0.0228	55.95	60.92
Yancheng	-0.0005	0.0243	-0.001	0.0262	49.81	37.77
Zhenjiang	0.0001	0.0217	0.0003	0.0218	56.43	68.71
Taizhou	-0.0014	0.0233	-0.0024	0.0231	52.41	83.98
Hefei	0.0006	0.0160	0.0011	NA	54.90	0
Wuhu	-0.0005	0.0181	-0.0003	NA	53.53	0
Ma'anshan	0.0005	0.0177	0.0009	NA	60.50	0
Anqing	0.0005	0.0204	0.0010	0.0214	53.83	32.18
Chuzhou	0.0002	0.0199	0.0004	NA	49.41	0
Xuancheng	-0.0005	0.0205	-0.0008	0.0204	53.75	10.30
Tongling	0.0012	0.0176	0.0020	NA	64.58	0
Chizhou	0.0010	0.0195	0.0014	0.0230	63.37	26.74

Table S4. Sensitivity analysis of NDVI and LST trends during BUA expansion across different urban development stages, excluding repeated city-stage transitions.

Stage	Cities	Average slope		Average significant slope		Proportion of significant change (%)	
		NDVI	LST	NDVI	LST	NDVI	LST
Stage 1*	1	-0.0017	0.0199	-0.0028	0.0252	46.53	1.44
Stage 2*	5	0.0005	0.0473	0.0031	0.0857	19.14	13.75
Stage 3*	5	-0.0007	0.0585	-0.0009	0.0863	24.73	25.08
Stage 4*	7	-0.0014	0.0370	-0.0021	0.0581	36.60	26.58
p for trends		0.022	0.264	0.022	0.072	0.029	0.662

Urban development stages are defined as follows: Stage 1 represents the transition from megacities to super megacities, comprising Shanghai. Stage 2* denotes the transition from large cities to megacities, including Hangzhou, Ningbo, Nanjing, Nantong and Wuxi. Stage 3* signifies the transition from medium to large cities, encompassing Taizhou (Zhejiang), Shaoxing, Changzhou, Yangzhou and Wuhu. Stage 4* captures the transition from small to medium cities, involving Jinhua, Yancheng, Jiaxing, Taizhou (Jiangsu), Huzhou, Ma'anshan, and Tongling. Note that some cities appear in multiple stages as they underwent consecutive transitions. Average slope: The mean value of Sen's slope estimates for all pixels in the analysis region. It represents the overall average rate of change in NDVI and LST over the study period; Significant average slope: The mean value of Sen's slope estimates only for pixels that passed the significance test ($p < 0.05$ in the Mann-Kendall test).

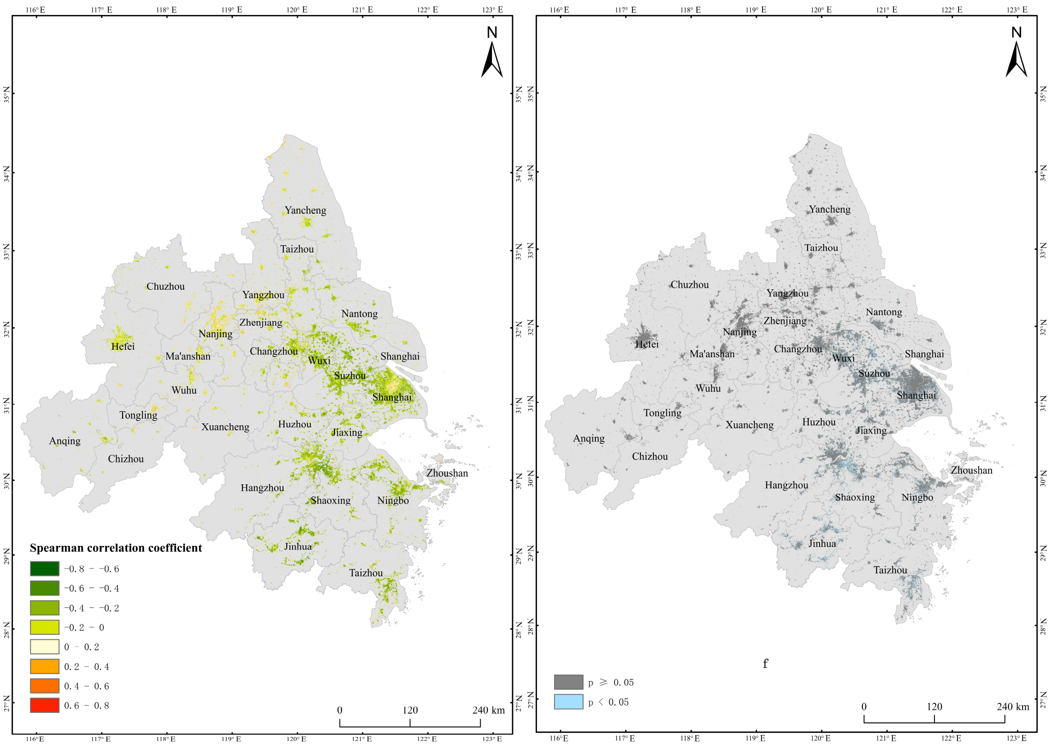

(a) (b)

Figure S1 Spatial distribution of Spearman correlation coefficients between annual NDVI and LST in the Yangtze River Delta. (a) Correlation coefficient; (b) statistical significance (two-tailed test, α = 0.05).
